# (4*R*)-4-[(1*R*)-1,2-Dihydroxy­ethyl]-1-[(1*R*)-1-phenyl­ethyl]pyrrolidin-2-one

**DOI:** 10.1107/S1600536808027293

**Published:** 2008-08-30

**Authors:** Adrian Blaser, Peter D. W. Boyd

**Affiliations:** a Cancer Research Laboratory, The University of Auckland, Private Bag 92019, Auckland, New Zealand; b Department of Chemistry, The University of Auckland, Private Bag 92019, Auckland, New Zealand

## Abstract

The title compound, C_14_H_19_NO_3_, was obtained as one of the two isomers of a Sharpless asymmetric dihydroxy­lation reaction of (1*S*)-1-[(1*R*)-1-phenyl­ethyl]-4-vinyl­pyrrolidin-2-one. The absolute stereochemistry of this isomer was determined from the known stereochemistry (*R*) at the bridge C atom between the phenyl and pyrrolidine rings. The mol­ecules form one-dimensional tapes along the *b* axis *via* hydrogen bonding between the carbonyl O atom and the alcohol groups of neighbouring mol­ecules. These assemble into sheets *via* inter­digitative stacking of the phenyl rings and C—H⋯O inter­actions.

## Related literature

For related literature see: Fava *et al.* (1999 ▶).
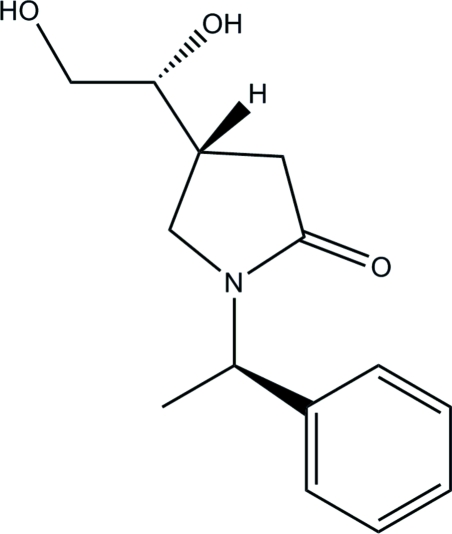


## Experimental

### 

#### Crystal data

C_14_H_19_NO_3_
*M* *_r_* = 249.30
Monoclinic, 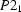

*a* = 6.1953 (1) Å
*b* = 8.2895 (2) Å
*c* = 13.2737 (1) Å
β = 103.353 (2)°
*V* = 663.25 (2) Å^3^
*Z* = 2
Mo *K*α radiation
μ = 0.09 mm^−1^
*T* = 83 (2) K
0.28 × 0.18 × 0.10 mm

#### Data collection

Siemens SMART APEX CCD diffractometer
Absorption correction: none
4016 measured reflections
1461 independent reflections
1271 reflections with *I* > 2σ(*I*)
*R*_int_ = 0.040

#### Refinement

*R*[*F* ^2^ > 2σ(*F* ^2^)] = 0.045
*wR*(*F* ^2^) = 0.099
*S* = 1.01
1461 reflections
166 parameters
1 restraint
H-atom parameters constrained
Δρ_max_ = 0.20 e Å^−3^
Δρ_min_ = −0.22 e Å^−3^

### 

Data collection: *SMART* (Siemens, 1995 ▶); cell refinement: *SAINT* (Siemens, 1995 ▶); data reduction: *SAINT*; program(s) used to solve structure: *SHELXS97* (Sheldrick, 2008 ▶); program(s) used to refine structure: *SHELXL97* (Sheldrick, 2008 ▶); molecular graphics: *ORTEPIII* (Burnett & Johnson, 1996 ▶) and *Mercury* (Macrae *et al.*, 2006 ▶); software used to prepare material for publication: *WinGX* (Farrugia, 1999 ▶).

## Supplementary Material

Crystal structure: contains datablocks I, global. DOI: 10.1107/S1600536808027293/at2618sup1.cif

Structure factors: contains datablocks I. DOI: 10.1107/S1600536808027293/at2618Isup2.hkl

Additional supplementary materials:  crystallographic information; 3D view; checkCIF report

## Figures and Tables

**Table 1:** Hydrogen-bond geometry (Å, °)

*D*—H⋯*A*	*D*—H	H⋯*A*	*D*⋯*A*	*D*—H⋯*A*
O3—H3⋯O1^i^	0.82	1.96	2.743 (3)	158
O2—H2⋯O1^i^	0.82	1.93	2.738 (3)	170

Symmetry code: (i) 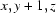.

**Fig. 1.**
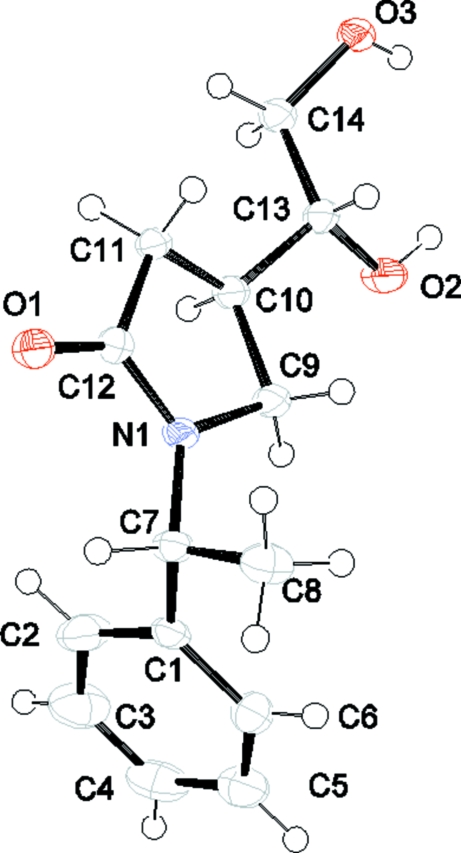
Structure of (I) showing 50% probability displacement ellipsoids for non-hydrogen atoms and hydrogen atoms as arbitary spheres.

**Fig. 2.**
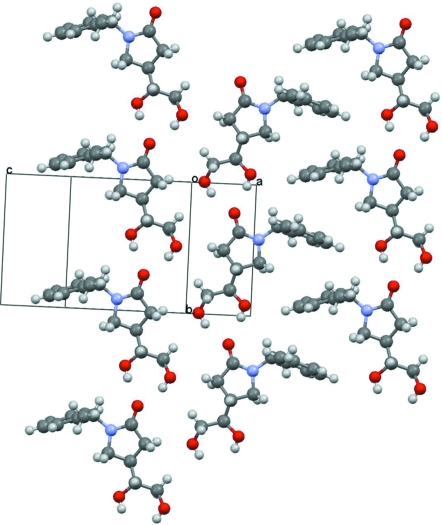
Illustration of the arrangement of (I) into sheets.

## supplementary crystallographic information

### Comment

The title compound (I) was obtained as one of the two isomers of a Sharpless asymmetric dihydroxylation reaction of (1*S*)-1-((1*R*)-1-phenylethyl)-4-vinylpyrrolidin-2-one (Fava *et al.*1999). The major isomer from the reaction was recrystallized to give a pure sample for X-ray analysis. The molecular structure of (I) is shown in Fig. 1. The assignment of the absolute stereochemistry is based on the known stereochemistry of C7 (*R*). This leads to the absolute configuration at C10 and C13 as *R*.

The molecules of (I) in the crystal form one dimensional tapes along the *b* axis *via* hydrogen bonding between the carbonyl oxygen, O1 and the two alcohol moieties O2—H and O3—H. These assemble by interdigitative stacking of phenyl rings between tapes and further connection by C11—H11B···O3, C5—H5···O2 interactions between adjacent molecules to form sheets near the b-c plane, Fig. 2 (Table 1).

### Experimental

(4*R*)-4-[(1*R*)-1,2-Dihydroxyethyl]-1-[(1*R*)-1-phenylethyl]-2-pyrrolidinone (I): AD-mix-β (1.40 g, 1 mmol, Aldrich Cat. No. 392766) was dissolved in *tert*-butanol (5 ml) and water (5 ml). Methanesulfonamide (98 mg, 1 mmol) was added and cooled to 273 K, (1*S*)-1-((1*R*)-1-phenylethyl)-4-vinylpyrrolidin-2-one1 (216 mg, 1 mmol) was added and the reaction stirred for 24 h. Na_2_SO_3_ (1.5 g, 11.9 mmol) was added and stirred for another 90 minutes. The reaction was extracted with dichloromethane (4x100 ml), the combined organic layers were washed with 2 N KOH solution, dried (Na_2_SO_4_) and concentrated under reduced pressure. The residue was purified by chromatography, eluting with methanol/ethylacetate (3:7) to give the two isomers (4*R*)-4-[(1*R*)-1,2-dihydroxyethyl]-1-[(1*R*)-1-phenylethyl]-2-pyrrolidinone and (4*R*)-4-[(1*S*)-1,2-dihydroxyethyl]-1-[(1*R*)-1-phenylethyl]-2-pyrrolidinone (169 mg, 68%) in a ratio of 2:1. The title compound (I) was then obtained by recrystallization from ethylacetate as clear crystals. ^1^H NMR (400 MHz, CDCl_3_) δ 7.36–7.24 (m, 5 H), 5.47 (q, J=7.1 Hz, 1 H), 3.69 (dd, J=10.7, 2.8 Hz, 1 H), 3.67–3.60 (m, 1 H), 3.46 (dd, J=10.7, 7.0 Hz, 1 H), 3.35 (dd, J=10.0, 7.0 Hz, 1 H), 3.13 (dd, J=10.0, 8.0 Hz, 1 H), 2.66 (br s, 1 H), 2.46 (dd, J=15.2, 8.1 Hz, 1 H), 2.40–2.29 (m, 1 H), 2.26 (dd, J=15.2, 8.2 Hz, 1 H), 2.17 (br s, 1 H), 1.53 (t, J=7.1 Hz, 3 H). LCMS (APCI^+^) calcd for C_14_H_19_NO_3_: 250 (MH^+^), found 250 (100%).

### Refinement

Hydrogen atoms were placed in calculated positions and refined using the riding model [O—H = 0.82 Å C—H = 0.93–0.97 Å], with *U*_iso_(H) = 1.5 times *U*_eq_(O) and *U*_iso_(H) = 1.2 or 1.5 times *U*_eq_(C)..

### Crystal data

**Table:**

C_14_H_19_NO_3_	*F*_000_ = 268
*M**_r_* = 249.30	*D*_x_ = 1.248 Mg m^−^^3^
Monoclinic, *P*2_1_	Mo *K*α radiation λ = 0.71073 Å
Hall symbol: P 2yb	Cell parameters from 2597 reflections
*a* = 6.1953 (1) Å	θ = 1.6–26.4º
*b* = 8.2895 (2) Å	µ = 0.09 mm^−^^1^
*c* = 13.2737 (1) Å	*T* = 83 (2) K
β = 103.353 (2)º	Plate, colourless
*V* = 663.25 (2) Å^3^	0.28 × 0.18 × 0.10 mm
*Z* = 2	

### Data collection

**Table:**

Siemens SMART APEX CCD diffractometer	1271 reflections with *I* > 2σ(*I*)
Radiation source: fine-focus sealed tube	*R*_int_ = 0.040
Monochromator: graphite	θ_max_ = 26.4º
*T* = 83(2) K	θ_min_ = 1.6º
ω scans	*h* = −7→7
Absorption correction: none	*k* = −10→10
4016 measured reflections	*l* = −7→16
1461 independent reflections	

### Refinement

**Table:**

Refinement on *F*^2^	Secondary atom site location: difference Fourier map
Least-squares matrix: full	Hydrogen site location: inferred from neighbouring sites
*R*[*F*^2^ > 2σ(*F*^2^)] = 0.045	H-atom parameters constrained
*wR*(*F*^2^) = 0.099	*w* = 1/[σ^2^(*F*_o_^2^) + (0.0168*P*)^2^ + 0.6853*P*] where *P* = (*F*_o_^2^ + 2*F*_c_^2^)/3
*S* = 1.01	(Δ/σ)_max_ < 0.001
1461 reflections	Δρ_max_ = 0.20 e Å^−^^3^
166 parameters	Δρ_min_ = −0.22 e Å^−^^3^
1 restraint	Extinction correction: none
Primary atom site location: structure-invariant direct methods	

### Special details

**Table:**

Geometry. All e.s.d.'s (except the e.s.d. in the dihedral angle between two l.s. planes) are estimated using the full covariance matrix. The cell e.s.d.'s are taken into account individually in the estimation of e.s.d.'s in distances, angles and torsion angles; correlations between e.s.d.'s in cell parameters are only used when they are defined by crystal symmetry. An approximate (isotropic) treatment of cell e.s.d.'s is used for estimating e.s.d.'s involving l.s. planes.
Refinement. Refinement of *F*^2^ against ALL reflections. The weighted *R*-factor *wR* and goodness of fit *S* are based on *F*^2^, conventional *R*-factors *R* are based on *F*, with *F* set to zero for negative *F*^2^. The threshold expression of *F*^2^ > σ(*F*^2^) is used only for calculating *R*-factors(gt) *etc*. and is not relevant to the choice of reflections for refinement. *R*-factors based on *F*^2^ are statistically about twice as large as those based on *F*, and *R*- factors based on ALL data will be even larger.

### Fractional atomic coordinates and isotropic or equivalent isotropic displacement parameters (Å^2^)

**Table:**

	*x*	*y*	*z*	*U*_iso_*/*U*_eq_	
O3	0.1980 (4)	−0.0064 (3)	0.00048 (16)	0.0261 (5)	
H3	0.2059	0.0736	−0.0348	0.039*	
O1	0.3278 (5)	−0.7778 (3)	−0.12286 (19)	0.0277 (6)	
O2	0.3068 (5)	−0.0804 (3)	−0.20720 (19)	0.0339 (6)	
H2	0.3289	0.0108	−0.1832	0.051*	
N1	0.4081 (4)	−0.5848 (3)	−0.2318 (2)	0.0200 (6)	
C11	0.2531 (5)	−0.4926 (4)	−0.0993 (2)	0.0204 (6)	
H11A	0.3554	−0.4685	−0.0339	0.024*	
H11B	0.1070	−0.5120	−0.0869	0.024*	
C7	0.5155 (5)	−0.6928 (4)	−0.2932 (2)	0.0216 (7)	
H7	0.4748	−0.8031	−0.2784	0.026*	
C13	0.3005 (6)	−0.1917 (4)	−0.1258 (3)	0.0231 (7)	
H13	0.4473	−0.1964	−0.0784	0.028*	
C9	0.4141 (6)	−0.4083 (4)	−0.2397 (3)	0.0231 (7)	
H9A	0.3682	−0.3731	−0.3112	0.028*	
H9B	0.5613	−0.3666	−0.2099	0.028*	
C12	0.3308 (5)	−0.6349 (4)	−0.1506 (2)	0.0216 (7)	
C1	0.4215 (5)	−0.6645 (4)	−0.4075 (2)	0.0222 (7)	
C8	0.7661 (5)	−0.6810 (5)	−0.2569 (3)	0.0320 (8)	
H8A	0.8137	−0.5754	−0.2721	0.048*	
H8B	0.8341	−0.7605	−0.2922	0.048*	
H8C	0.8089	−0.6998	−0.1837	0.048*	
C10	0.2466 (6)	−0.3545 (4)	−0.1767 (3)	0.0217 (7)	
H10	0.0983	−0.3499	−0.2228	0.026*	
C6	0.5480 (6)	−0.6089 (4)	−0.4737 (2)	0.0297 (8)	
H6	0.6968	−0.5842	−0.4474	0.036*	
C14	0.1299 (6)	−0.1421 (4)	−0.0656 (2)	0.0236 (7)	
H14A	−0.0084	−0.1162	−0.1142	0.028*	
H14B	0.1024	−0.2326	−0.0240	0.028*	
C4	0.2361 (7)	−0.6207 (6)	−0.6180 (3)	0.0433 (10)	
H4	0.1743	−0.6056	−0.6882	0.052*	
C2	0.2008 (6)	−0.6969 (6)	−0.4493 (3)	0.0395 (10)	
H2A	0.1129	−0.7347	−0.4063	0.047*	
C5	0.4561 (7)	−0.5893 (5)	−0.5789 (3)	0.0370 (9)	
H5	0.5444	−0.5548	−0.6227	0.044*	
C3	0.1060 (7)	−0.6746 (7)	−0.5538 (3)	0.0491 (12)	
H3A	−0.0438	−0.6959	−0.5801	0.059*	

### Atomic displacement parameters (Å^2^)

**Table:**

	*U*^11^	*U*^22^	*U*^33^	*U*^12^	*U*^13^	*U*^23^
O3	0.0388 (14)	0.0189 (12)	0.0245 (11)	−0.0048 (12)	0.0151 (10)	−0.0050 (11)
O1	0.0460 (16)	0.0153 (12)	0.0264 (13)	−0.0007 (11)	0.0178 (12)	0.0004 (10)
O2	0.0633 (18)	0.0161 (12)	0.0291 (13)	−0.0033 (14)	0.0247 (13)	−0.0008 (10)
N1	0.0294 (15)	0.0130 (13)	0.0208 (13)	−0.0012 (12)	0.0121 (11)	−0.0019 (11)
C11	0.0293 (16)	0.0170 (15)	0.0176 (14)	−0.0016 (15)	0.0111 (12)	−0.0007 (14)
C7	0.0299 (17)	0.0168 (17)	0.0202 (15)	0.0016 (15)	0.0103 (13)	−0.0010 (13)
C13	0.0341 (18)	0.0166 (16)	0.0219 (16)	−0.0032 (15)	0.0135 (14)	−0.0008 (14)
C9	0.035 (2)	0.0168 (16)	0.0211 (16)	−0.0030 (15)	0.0134 (14)	−0.0025 (14)
C12	0.0269 (17)	0.0193 (16)	0.0196 (15)	−0.0028 (14)	0.0071 (13)	−0.0005 (14)
C1	0.0292 (17)	0.0180 (17)	0.0203 (15)	0.0016 (14)	0.0073 (13)	−0.0056 (14)
C8	0.0330 (19)	0.038 (2)	0.0248 (17)	0.0042 (18)	0.0050 (14)	−0.0050 (16)
C10	0.0309 (19)	0.0178 (16)	0.0191 (16)	−0.0010 (15)	0.0112 (14)	−0.0004 (13)
C6	0.041 (2)	0.0249 (19)	0.0255 (17)	−0.0037 (17)	0.0125 (15)	0.0010 (16)
C14	0.0327 (18)	0.0182 (16)	0.0225 (15)	−0.0025 (14)	0.0116 (13)	−0.0022 (14)
C4	0.059 (3)	0.044 (3)	0.0239 (17)	0.014 (2)	0.0029 (17)	−0.0049 (18)
C2	0.034 (2)	0.060 (3)	0.0269 (18)	−0.003 (2)	0.0112 (15)	−0.011 (2)
C5	0.063 (3)	0.0251 (19)	0.0269 (18)	0.001 (2)	0.0178 (18)	0.0022 (16)
C3	0.035 (2)	0.075 (4)	0.034 (2)	0.002 (2)	0.0001 (17)	−0.017 (2)

### Geometric parameters (Å, °)

**Table:**

O3—C14	1.429 (4)	C9—H9A	0.9700
O3—H3	0.8200	C9—H9B	0.9700
O1—C12	1.242 (4)	C1—C2	1.378 (5)
O2—C13	1.428 (4)	C1—C6	1.385 (4)
O2—H2	0.8200	C8—H8A	0.9600
N1—C12	1.342 (4)	C8—H8B	0.9600
N1—C9	1.468 (4)	C8—H8C	0.9600
N1—C7	1.469 (4)	C10—H10	0.9800
C11—C12	1.496 (5)	C6—C5	1.390 (5)
C11—C10	1.532 (4)	C6—H6	0.9300
C11—H11A	0.9700	C14—H14A	0.9700
C11—H11B	0.9700	C14—H14B	0.9700
C7—C1	1.513 (4)	C4—C5	1.367 (6)
C7—C8	1.519 (5)	C4—C3	1.376 (6)
C7—H7	0.9800	C4—H4	0.9300
C13—C10	1.511 (5)	C2—C3	1.388 (5)
C13—C14	1.521 (4)	C2—H2A	0.9300
C13—H13	0.9800	C5—H5	0.9300
C9—C10	1.542 (5)	C3—H3A	0.9300
			
C14—O3—H3	109.5	C6—C1—C7	123.0 (3)
C13—O2—H2	109.5	C7—C8—H8A	109.5
C12—N1—C9	112.7 (3)	C7—C8—H8B	109.5
C12—N1—C7	123.2 (3)	H8A—C8—H8B	109.5
C9—N1—C7	123.1 (3)	C7—C8—H8C	109.5
C12—C11—C10	104.2 (2)	H8A—C8—H8C	109.5
C12—C11—H11A	110.9	H8B—C8—H8C	109.5
C10—C11—H11A	110.9	C13—C10—C11	113.5 (3)
C12—C11—H11B	110.9	C13—C10—C9	113.2 (3)
C10—C11—H11B	110.9	C11—C10—C9	103.3 (3)
H11A—C11—H11B	108.9	C13—C10—H10	108.9
N1—C7—C1	110.1 (3)	C11—C10—H10	108.9
N1—C7—C8	110.3 (3)	C9—C10—H10	108.9
C1—C7—C8	115.9 (3)	C1—C6—C5	121.0 (3)
N1—C7—H7	106.7	C1—C6—H6	119.5
C1—C7—H7	106.7	C5—C6—H6	119.5
C8—C7—H7	106.7	O3—C14—C13	113.2 (3)
O2—C13—C10	106.3 (3)	O3—C14—H14A	108.9
O2—C13—C14	111.5 (3)	C13—C14—H14A	108.9
C10—C13—C14	111.6 (3)	O3—C14—H14B	108.9
O2—C13—H13	109.1	C13—C14—H14B	108.9
C10—C13—H13	109.1	H14A—C14—H14B	107.8
C14—C13—H13	109.1	C5—C4—C3	120.1 (4)
N1—C9—C10	102.6 (3)	C5—C4—H4	119.9
N1—C9—H9A	111.2	C3—C4—H4	119.9
C10—C9—H9A	111.2	C1—C2—C3	121.9 (4)
N1—C9—H9B	111.2	C1—C2—H2A	119.1
C10—C9—H9B	111.2	C3—C2—H2A	119.1
H9A—C9—H9B	109.2	C4—C5—C6	120.0 (3)
O1—C12—N1	124.5 (3)	C4—C5—H5	120.0
O1—C12—C11	126.1 (3)	C6—C5—H5	120.0
N1—C12—C11	109.5 (3)	C4—C3—C2	119.3 (4)
C2—C1—C6	117.6 (3)	C4—C3—H3A	120.4
C2—C1—C7	119.3 (3)	C2—C3—H3A	120.4

### Hydrogen-bond geometry (Å, °)

**Table:**

*D*—H···*A*	*D*—H	H···*A*	*D*···*A*	*D*—H···*A*
O3—H3···O1^i^	0.82	1.96	2.743 (3)	158
O2—H2···O1^i^	0.82	1.93	2.738 (3)	170

Symmetry codes: (i) *x*, *y*+1, *z*.
